# Chromosome-level genome assembly of Korean native cattle and pangenome graph of 14 *Bos taurus* assemblies

**DOI:** 10.1038/s41597-023-02453-z

**Published:** 2023-08-23

**Authors:** Jisung Jang, Jaehoon Jung, Young Ho Lee, Sanghyun Lee, Myunggi Baik, Heebal Kim

**Affiliations:** 1 Interdisciplinary Program in Bioinformatics, Seoul National University, Seoul, Republic of Korea; 2 Department of Agricultural Biotechnology and Research Institute of Agriculture and Life Sciences, College of Agriculture and Life Sciences, Seoul National University, Seoul, Korea

**Keywords:** Genomics, Genome

## Abstract

This study presents the first chromosome-level genome assembly of Hanwoo, an indigenous Korean breed of *Bos taurus taurus*. This is the first genome assembly of Asian taurus breed. Also, we constructed a pangenome graph of 14 *B. taurus* genome assemblies. The contig N50 was over 55 Mb, the scaffold N50 was over 89 Mb and a genome completeness of 95.8%, as estimated by BUSCO using the mammalian set, indicated a high-quality assembly. 48.7% of the genome comprised various repetitive elements, including DNAs, tandem repeats, long interspersed nuclear elements, and simple repeats. A total of 27,314 protein-coding genes were identified, including 25,302 proteins with inferred gene names and 2,012 unknown proteins. The pangenome graph of 14 *B. taurus* autosomes revealed 528.47 Mb non-reference regions in total and 61.87 Mb Hanwoo-specific regions. Our Hanwoo assembly and pangenome graph provide valuable resources for studying *B. taurus* populations.

## Background & Summary

Hanwoo is a native Korean taurine cattle breed with a 5000-year history as a draft animal for farming and transportation^1^. In a short period, Hanwoo underwent significant changes in its demographic history and selection. During the Korean war (1950–1953), the number of Hanwoo dropped to about 390,000, but recovered to 1.02 million by the late 1950s. With the development of the South Korean economy and agricultural industry, Hanwoo transitioned from a draft to a meat production breed in the 1960s. Modern breeding programs, including performance tests, artificial insemination and genomic selection were initiated by the South Korean government in the 1980s. These programs have improved carcass weight and meat quality of Hanwoo by increasing intramuscular fat (marbling). As a result of continuous artificial selection, Hanwoo has gained unique features both in genome and traits.

This study presents a high-quality assembly of Hanwoo which is the first chromosome-level genome assembly of Asian *Bos taurus taurus* using a combination of PacBio Hifi, Isoform and Illumina RNA sequencing, with scaffold N50 length of 89 Mb. The completeness of the genome was confirmed by the BUSCO score of 95.8%. The top 31 scaffolds are all greater than 17 Mb in size with a total length of 2.69 Gb. 48.7% of the Hanwoo genome is composed of various repetitive elements. The genome was annotated to contain 27,314 protein-coding genes, including 25,302 proteins with inferred gene names and 2,012 unknown proteins.

We generated a pangenome graph of 14 high-quality *Bos taurus* autosomes including high-quality genome assemblies of Hanwoo, Hereford, Angus, Brown Swiss, Highland, Holstein, Jersey, Original Braunvieh, Piedmontese, Simmental, Brahman, Nellore, N’Dama, and Ankole. We identified non-reference regions and breed-specific regions through the pangenome graph. In Hanwoo, 528.47 Mb of total non-reference nodes and 61.87 Mb of Hanwoo-specific nodes were identified. This pangenome graph would be used to extract structural variations and make insightful observations among various populations of *Bos taurus*.

## Methods

### Sample collection and extraction of genomic DNA and RNA

**Fig. 1:**
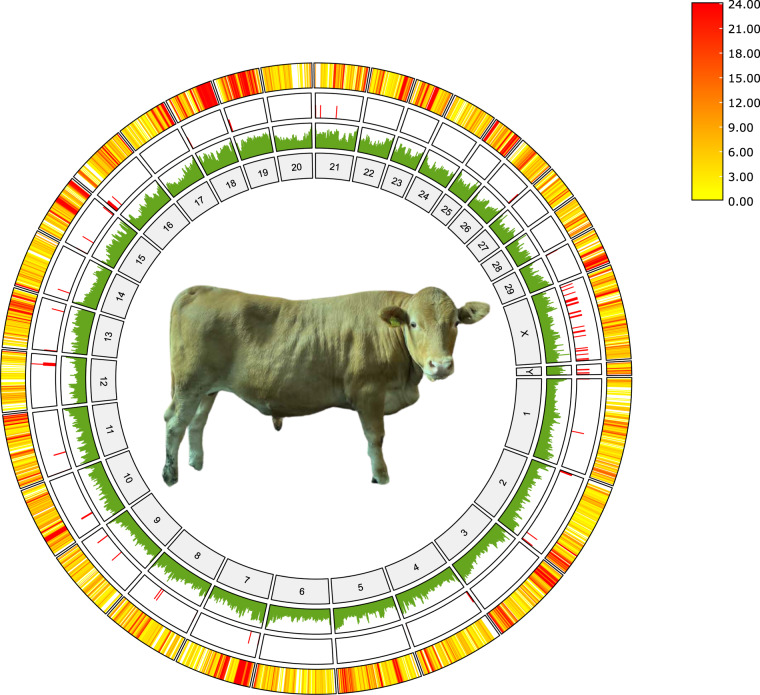
Picture of Hanwoo steer used in this study and a circos plot. Shown from the outer to inner circle are the following: gene density, with the intensity of color representing the number of genes in a 10,000 bp window; N (unknown base) ratio, with the height of the bar representing the percentage of bases that are N in a 1,000,000 bp window and the overall height of the track representing from the minimum to maximum value for the whole genome which are from 0% to 0.02%, respectively; GC content, with the height of the bar representing the percentage of GC in a 10,000 bp window and the overall height of the track representing from the minimum to maximum value for the whole genome which are from 27.07% to 74.80%, respectively; and the corresponding chromosome.

The samples used in the study of Hanwoo genome included blood, sirloin, liver, and subcutaneous fat from a steer named “bull 2050”. The samples were collected from the Experimental farm of College of Agriculture and Life Sciences at Seoul National University, Pyeongchang-gun, Gangwon-do, Republic of South Korea (Fig. 1) and were approved by the Seoul National University Institutional Animal Care and Use Committee (SNU-201129-1-1). It was castrated in 9.4 months of age, slaughtered and sampled in 32 months of age. All blood sampling was carried out by trained veterinarians, according to the approved institutional protocols. Genomic DNA were extracted from whole blood using Wizard Genomic DNA Purification kit following the manufacturer’s protocol.

Sirloin, liver and subcutaneous fat tissues of Hanwoo bull 2050 were collected immediately after slaughter and frozen using liquid nitrogen and stored in a deep freezer until RNA extraction. RNA was isolated using the RNeasy kits (Qiagen, Valencia, CA) following the manufacturer’s protocol.

### DNA library construction and sequencing

DNA sequencing libraries were prepared using SMRTbell Express Template Prep kit 2.0 (Pacific Biosciences, California, USA) and libraries larger than 20 kb were used for next steps. HiFi reads were sequenced using 2 SMRT cells of 8 M Tray, Sequel II Sequencing Kit 2.0 in Pacific Biosciences (PacBio) Sequel IIe platform at NICEM in Seoul National University. Highly accurate consensus sequences were produced by PacBio *CCS* workflow (v 6.3.0), yielding a total of 3.5 M reads and 67.5Gbp corresponding to a genomic coverage of ~24.8X (Table 1).

**Table 1:** Statistics of sequencing data.

Platform	Tissue	Reads	Total bases (bp)	Average length (bp)	N50 length (bp)	SRA accession
PacBio	Blood	3,520,375	67,520,132,790	19180	20224	SRR23238456
RNA-seq	Liver	37986259	5773911368	76	76	SRR23238454
	Subcutaneous fat	37619668	5718189536	76	76	SRR23238453
	Sirloin	40572880	6167077760	76	76	SRR23238455
Iso-Seq	Sirloin	10,054,509	20,639,745,850	2,052	2,268	SRR23238452

### RNA library construction and sequencing

For RNA-seq, paired-end libraries with insert size of 75 bp were prepared with TruSeq Stranded mRNA Sample Preparation kit (Illumina, San Diego CA USA) from total messenger RNA (mRNA) of sirloin, liver and subcutaneous fat tissues of a Hanwoo bull 2050. RNA of the three tissues were sequenced separately using Illumina NextSeq 500 with following adapters; liver: D701, D506; sirloin: D701, D507; subcutaneous fat: D701, D508. 17.65 Gb of short paired-end RNA reads were sequenced using Illumina NextSeq 500 (Table 1).

For Iso-Seq, a total of 600 ng RNA from sirloin was used for full-length transcript sequencing with Pacbio Sequel system (Pacific Biosciences, CA, USA) according to the manufacturer’s instructions. The Iso-Seq library was prepared according to the Isoform Sequencing (Iso-Seq) protocol using the NEBNext Single Cell/Low Input cDNA Synthesis & Amplification Module, PacBio SMRTbell Express Template Prep Kit 2.0 and ProNex® Size-Selective Purification System.

Total 10 μL library was prepared using PacBio SMRTbell Express Template Prep Kit 2.0. SMRTbell templates were annealed using Sequel Binding and Internal Ctrl Kit 3.0. The Sequel Sequencing Kit 3.0 and SMRT cells 1 M v3 LR Tray was used for sequencing. SMRT cells (Pacific Biosciences) using 1200 min movies were captured for each SMRT cell using the PacBio Sequel System (Pacific Biosciences).

### Genome size estimation and contig assembly

**Fig. 2:**
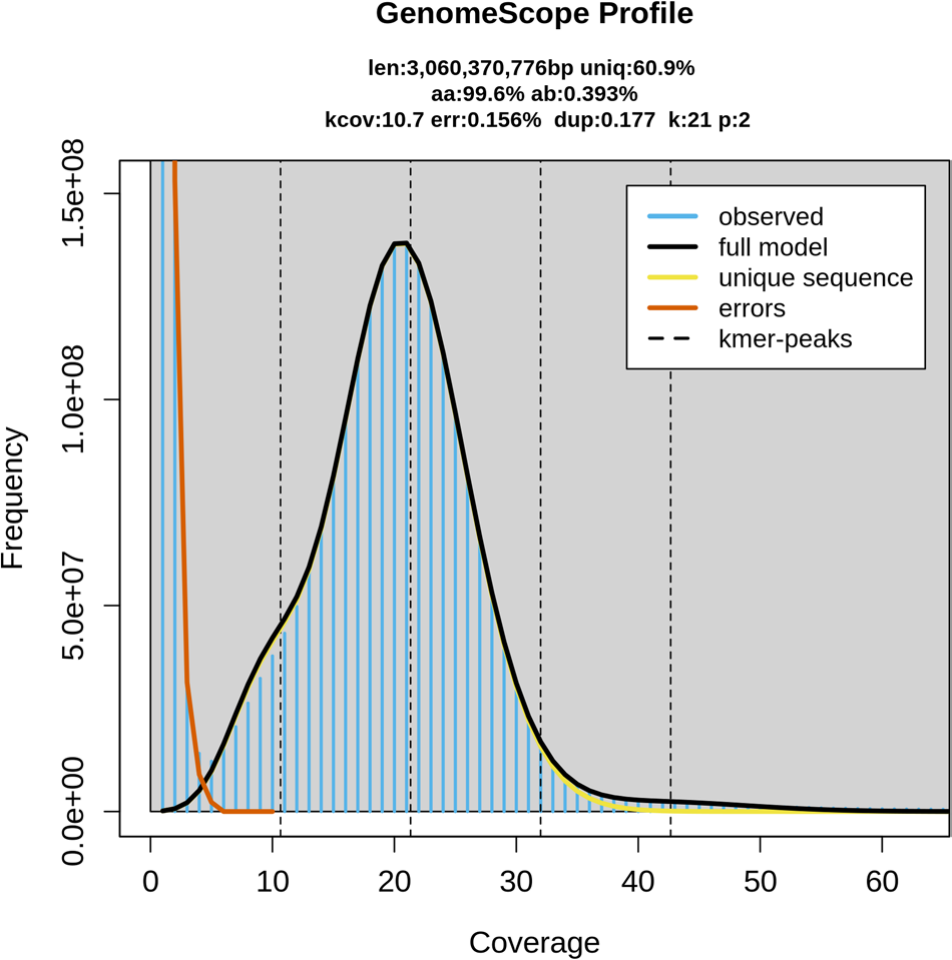
Genome size estimation by GenomeScope2.

Hanwoo contigs were assembled using the HiFi consensus reads and validated following the VGP (Vertebrate Genomes Project) assembly pipeline^2^. Adapter sequences of HiFi reads (5′–ATCTCTCTCTTTTCCTCCTCCTCCGTTGTTGTTGTTGAGAGAGAT–3′) were removed by Cutadapt (v 4.0)^3^. Counting *k*-mer and generating histogram of the *k*-mer count were performed on adapter trimmed sequences with *k* = 21 by Meryl (v 1.3.0)^4^. Genome properties such as genome size, maximum read depth and transition parameter were inferred using GenomeScope (v 2.0)^5^ from the 21-mer histogram generated by Meryl (v 1.3.0)^4^. Genome size of Hanwoo was estimated as 3.06 Gb based on the *k*-mer histogram (Fig. 2). Trimmed reads were assembled to contig level using Hifiasm (v 0.16.1)^6^, and the draft contig assembly consisted of 1311 contigs totaling 3.28 Gb with an N50 of 55.23 Mb (Table 2). Haplotypic duplication and low-coverage contigs of the draft contig assembly were removed using Purge_dups (v 1.2.5)^7^ after self-alignment using Minimap2^8^. The primary contig assembly after removing haplotypic duplication included 603 contigs, with a size of 3.11 Gb and a contig N50 of 58.14 Mb.

**Table 2:** Statistics of contig assembly before scaffolding.

Statistics without reference	Draft primary contig assembly	Draft alternate contig assembly	Purged primary contig assembly	Purged alternate contig assembly
Number of contigs	1311	12491	603	8339
Largest contig	154270589	4548020	154270589	4727895
Total length	3278632171	2786845933	3108034269	2585754757
N50	55230564	664031	58141786	746831
N75	15456913	255609	23975184	356899
L50	19	1163	18	998
L75	44	2817	37	2240
GC (%)	43.93	43.81	43.44	43.13

### Scaffolding and gap filling

The Hanwoo contigs after removing haplotypic duplication were scaffolded on autosome of *ARS-UCD1.3*, through reference-guided approach by RagTag (v 2.1.0)^9^. Because the Y chromosome is absent in *ARS-UCD1.3*, autosome and X chromosome of *ARS-UCD1.3*, and Y chromosome of *UOA_Angus_1* were used as reference genome for scaffolding. The reference-guided scaffolding using RagTag (v 2.1.0)^9^ consist of ‘correct’ and ‘scaffold’ steps. The ‘correct’ step identified and corrected potential misassembly based on alignment of contig assembly to the reference genome assembly. Part of contigs were broken at points of putative misassembly, and as a result, the number of contigs increased to 1915. In the ‘scaffold’ step, these RagTag ‘corrected’ contigs were aligned to the reference genome consist of autosome and X chromosome of *ARS-UCD1.3*, and Y chromosome of *UOA_Angus_1*. As a result, there were 1598 scaffolds including 31 chromosome-level scaffolds and 1567 unplaced scaffolds.

HiFi reads used in the Hanwoo assembly were aligned using Minimap2^8^ to perform gap filling of the chromosome-level Hanwoo genome assembly using TGS-GapCloser (v 1.0.1)^10^. The final 31 chromosome-level scaffolds had a total size of 2.69 Gb, which was similar to chromosome size of *ARS-UCD 1.3*. (Tables 3, 4). These 31 chromosome-level scaffolds composed 86.66% of the assembly, with the remaining 414.6 Mb still unanchored and requiring further investigation. Further analysis including annotation and pangenome were performed on the chromosome-level scaffolds.

**Table 3:** Hanwoo genome assembly statistics.

Assembly statistics	Value
Genome size (bp)	3108492884
Number of scaffolds	1598
Number of chromosome-scale scaffolds	31
N50 of scaffolds (bp)	89243566
L50 of scaffolds	13
Chromosome-scale scaffolds (bp)	2693904935
GC content of the genome (%)	43.44
QV score	63.68
Error rate	4.29E-07
BUSCO analysis	
Library	mammalia_odb10
Complete	8842 (95.8%)
Complete and single copy	8664 (93.9%)
Complete and duplicated	178 (1.9%)
Fragmented	106 (1.1%)
Missing	278 (3.1%)

**Table 4:** Length of Chromosome-level scaffolds.

Chromosome	Length	% of assembly
1	158347075	5.88
2	140532406	5.22
3	121557778	4.51
4	122787172	4.56
5	121175501	4.50
6	120343135	4.47
7	111272195	4.13
8	114613683	4.25
9	105990968	3.93
10	104650420	3.88
11	107792557	4.00
12	89243566	3.31
13	85553472	3.18
14	83497117	3.10
15	85308379	3.17
16	88665756	3.29
17	73790049	2.74
18	68766244	2.55
19	65427893	2.43
20	71637878	2.66
21	78435670	2.91
22	61025439	2.27
23	53933626	2.00
24	63313671	2.35
25	42768661	1.59
26	53441352	1.98
27	46802419	1.74
28	46008137	1.71
29	52029189	1.93
X	137682877	5.11
Y	17510650	0.65
Total	2693904935	100.00

Circos plot denoting gene density, N ratio and GC content was generated with the advanced circos function from Java-based tool TBtools^11^. The gene density (number of genes), N ratio (%) and GC content (%) was calculated for every 10,000 bp increment of the genome and was visualized in a heatmap format for gene density and histogram format for N ratio and GC content using BIN size 100,000.

### Masking repetitive sequences

Repetitive sequences in the gap-filled Hanwoo assembly were soft-masked using RepeatMasker (v 4.1.5)^12^ with a known library (cow) in Dfam (v 3.7) and RepBase (v 10/26/2018) using RMBlast. Repetitive elements predicted by RepeatMasker contained 1.31 Gb of sequences, accounting for 48.7% of the genome, including 27.6%, 11.6%, 4.9%, 2.1% and 1.5% for LINEs, SINEs, LTR elements, DNA elements, and satellite repeats, respectively (Table 5).

**Table 5:** Statistics of repetitive elements.

Class	Subclass	Number	Total length (bp)	% of genome
SINEs:		2083225	312596265	11.6
	MIRs	399931	57592626	2.14
LINEs:		1318367	742600414	27.57
	LINE1	584926	340390426	12.64
	LINE2	255007	65643056	2.44
	L3/CR1	34731	7189988	0.27
	RTE	442538	329203364	12.22
LTR elements:		415490	131192082	4.87
	ERVL	75217	29646401	1.1
	ERVL-MaLRs	121580	39874562	1.48
	ERV_classI	84207	37072606	1.38
	ERV_classII	117558	20606823	0.76
DNA elements:		289836	57547635	2.14
	hAT-Charlie	163969	30537889	1.13
	TcMar-Tigger	45005	11907379	0.44
Unclassified:		3023	464793	0.02
Total interspersed repeats:			1244401189	46.19
Small RNA:		254380	43115368	1.6
Satellites:		6216	39399744	1.46
Simple repeats:		537045	22458650	0.83
Low complexity:		81860	4022678	0.15
Total bases masked:			1311158349	48.67

### Genome annotation

Illumina RNA-seq reads were trimmed to remove adapter sequences and low-quality bases using Trimmomatic (v 0.39)^13^. The BRAKER3 (v 3.0.3) pipeline was used for structural annotation of Hanwoo genome. The pipeline utilized three sources of extrinsic evidence; short-read RNA-seq (Illumina), protein sequences of Vertebrata in OrthoDB (v 11)^14^ in addition to protein sequence of *ARS-UCD1.3* to train Augustus (v 3.5.0)^15^ for gene prediction.

The predicted gene sets were searched in 2 public functional databases, Swiss-Prot of UniProtKB^16^ and Pfam (v 35.0) database^17^ to identify the potential function with BLASTP (v 2.13.0+)^18^ and functional domains with InterProScan (v 5.57)^19^. We used scripts included in MAKER (v 3.01.03)^20^ to integrate functional annotations into structural annotations. The protein annotation was evaluated by analyzing amino acid sequences of protein using BUSCO (v 5.3.2)^21^ with the conserved core set of mammalian genes, yielding a completeness score of 87.9%. A total of 27,314 protein-coding genes were identified, including 25,302 genes with inferred names and 2,012 unknown proteins.

### Assessment of the chromosome-level genome assembly

N50, L50 and lengths of the chromosome-level Hanwoo genome assembly was calculated by QUAST (v 5.0.2)^22^. Single copy gene completeness was assessed with BUSCO (v 5.3.2)^21^, using the metaeuk backend against ‘mammalia_odb10’. Quality values (QV) was calculated with Merqury (v 1.3)^23^, with *k*-mer databases (*k* = 21) constructed by Meryl (v 1.3)^4^.

### Pangenome graph construction

The pangenome graph of 14 Bos taurus genomes, including the Hanwoo assembly, was generated using the Minigraph-Cactus Pangenome Pipeline (v 2.5.2)^24^. 14 assemblies were collected with the Hereford assembly, *ARS-UCD1.3*^25^, as the reference genome. 8 haplotype-resolved assemblies of Angus (*UOA_Angus_1*, GCF_002263795.3), Brahman (*UOA_Brahman_1*)^26^, Simmental (*ARS-Simm1.0*)^27^, Scottish Highland bull (*ARS_UNL_Btau-highland_paternal_1.0_alt*, GCA_009493655.1)^28^, N’Dama (*ROSLIN_BTT_NDA1*), Ankole (*ROSLIN_BTI_ANK1*)^29^, Jersey (*ARS-LIC_NZ_Jersey*, GCA_021234555.1), Holstein Friesian (*ARS-LIC_NZ_Holstein-Friesian_1*, GCA_021347905.1) were obtained from NCBI. Original Braunvieh^30^, Nellore, Brown Swiss, and Piedmontese were collected from the public database (10.5281/ZENODO.5906579) and scaffolded and merged by RagTag^9^ following the protocol of previous article^31^. The repeat sequences in the genomes of Original Braunvieh, Nellore, Brown Swiss, Piedmontese and Highland were soft-masked for by RepeatMasker (v 4.1.5)^12^ using same parameters and repeat databases with Hanwoo. Because one sex chromosome was missing in haplotype-resolved genomes produced by trio-binning assembly, only autosomes were included in our pangenome graph.

The Minigraph-Cactus Pangenome Pipeline consisted of four steps: constructing the Minigraph GFA, mapping the genomes back to the Minigraph, creating the Cactus alignment and creating the VG indexes. The Minigraph graph was created using ARS-UCD1.3 as the reference genome, and the other 13 genomes were iteratively added. Base-level alignments of the genomes were added to the graph using Cactus^24^. After embedding the haplotypes into the graph, Cactus alignment were performed, resulting in variation graph (VG) and hierarchical alignment (HAL). The HAL file was converted to packed graph (PG) and chopped into 32 base pairs using ‘hal2vg’ to describe it as nodes and edges.

### Non-reference nodes in pangenome graph

**Fig. 3:**
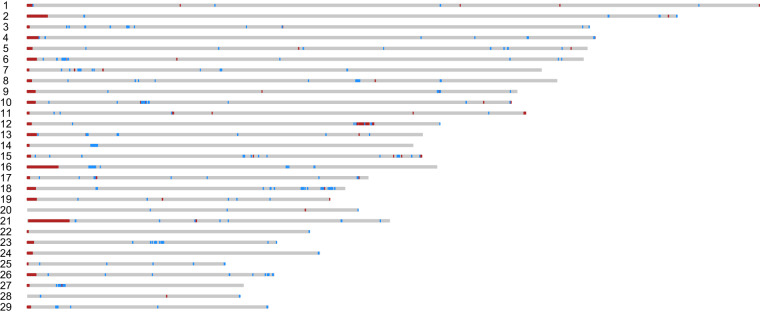
Non-reference region and specific region in Hanwoo autosome. Non-reference regions and Hanwoo-specific regions larger than or equal to 10 kb are visualized on Hanwoo autosomes. The Hanwoo-specific regions are marked in red, while the non-reference regions shared by other *Bos taurus* assemblies, excluding the Hanwoo-specific regions, are marked in blue.

The multiple whole-genome alignments generated by CACTUS^24^ were transformed into the Packed Graph (PG) format by chopping into 32 base pairs using ‘hal2vg’ with the options ‘—chop 32’ and ‘—noAncestors’^32^. The reference nodes and non-reference nodes were separated using scripts from the Github repository (https://github.com/evotools/CattleGraphGenomePaper/tree/master/detectSequences/nf-GraphSeq) following previous research^29^. After excluding nodes flanking with gaps in 1 kb, the counts and lengths of the non-reference and breed-specific nodes were calculated (Table 6). Non-reference region and Hanwoo-specific regions longer and equal to 10 kb are marked in Hanwoo autosome using KaryoploteR^33^ (Fig. 3). The Hanwoo-specific regions are encompassed within the non-reference region, with the majority of these regions being located in the telomeric and centromeric regions. Notably, the size of satellite repeats, as identified by RepeatMasker, amounted to 39.4 Mb (Table 5). The total size of the satellite repeat, a main component of the centromere, were similar to the differences in autosome length between Hanwoo and others. This finding implies that the larger genome and specific region of Hanwoo can be attributed to expansions within repeat-rich telomeric and centromeric regions.

**Table 6:** Sequence contribution of 14 bos taurus autosomes.

Breed	Non-reference nodes		Specific nodes		Total length (autosome)
	nodes	bp	nodes	bp	bp
Hanwoo	5644829	83917034	622052	61869953	2538711408
Angus	4876028	40793146	331609	23589072	2468157877
Brown Swiss	5135844	25626114	364958	8631263	2497220059
Highland	4917533	32014564	383674	14515221	2483452092
Holstein	5046695	31095517	434031	16204587	2468170459
Jersey	5050922	27795391	402709	11095169	2473656513
Original Braunvieh	5135877	27234395	361737	10537892	2503654516
Piedmontese	5128788	28520430	389915	11411557	2500499917
Simmental	5266669	40554393	527318	20773580	2494093306
Brahman	11480493	46633118	2650315	20140251	2478073158
Nellore	12648594	45129061	3423881	19092260	2502536439
N’Dama	7225426	54175845	1375922	35064951	2504036093
Ankole	8960222	44980693	1959559	23916971	2485084605
Hereford					2489385779

Furthermore, HiFi-based assemblies generally have higher telomeric completeness than Oxford nanopore- or CLR-based assemblies^34^. The uniqueness of origin and evolution history also supported the larger and disctinct genome of Hanwoo compared to European taurine. Mitochondrial DNA haplogroup of Hanwoo is P, which is common in European aurochs but has not been detected in modern cattle in Europe^35^. The haplogroup P mtDNA in Hanwoo suggested the possibility of a minor and local event of domestication or introgression of Asian aurochs^36,37^. Furthermore, intensive inbreeding and small effective population size of Hanwoo might facilitate fixation of these distinctive regions in Hanwoo genome^38^.

## Data Records

The final genome assembly was deposited at DDBJ/ENA/GenBank under the accession JARDUZ000000000^39^.

This Whole Genome Shotgun project has been deposited at DDBJ/ENA/GenBank under the accession SRR23238456^40^.

The transcriptomic Illumina sequencing data of subcutaneous fat, liver and sirloin were deposited in the SRA at NCBI SRR23238453, SRR23238454 and SRR23238455, respectively^40^.

The transcriptomic PacBio sequencing data of sirloin were deposited in the SRA at NCBI SRR23238452^40^.

The Hanwoo genome assembly which were not processed by NCBI, genome annotation, transcript sequence and protein sequence are available in figshare^41^.

The pangenome graph in GFA format are also available in figshare^42^.

## Technical Validation

RNA degradation and contamination were monitored on Agilent RNA ScreenTape. The purity of RNA samples was checked using the NanoPhotometer spectrophotometer (IMPLEN, CA, USA). The integrity of RNA was assessed using the RNA ScreenTape of the Agilent 2200 TapeStation System (Agilent Technologies, CA, USA). Only RNAs with an OD260/280 ratio of 2.0–2.2, an OD260/230 ratio of 1.8–2.1, and a RIN value of ≥9.0 were considered qualified for use. RNA concentration was measured using Quant-iT™ RiboGreen™ RNA Assay Kit in Victor Nivo (PerkinElmer, Waltham, MA, USA).

The completeness of the Hanwoo genome assembly was evaluated using BUSCO^21^ with the mammalian data set “mammalia_odb10.” The evaluation found 95.8% (8842) of the core mammalian genes were present in the genome, including 93.9% single-copy, 1.9% duplicated, 1.9% fragmental, and 3.1% missing genes from the mammalian data set (Table 3). The *k*-mer databases (*k* = 21) constructed using HiFi reads by Meryl^4^, and the overall assembly quality was assessed using the *k*-mer databases using Merqury^23^. The assembly showed high quality values (QV > 63) with an error rate of 4.29 × 10^−7^ (Table 3). The GC content of Hanwoo (43.44%) was slightly higher than that of *ARS-UCD1.3* (41.56%). These assessment results confirmed the completeness of Hanwoo genome assembly (Table 3).

## Data Availability

Parameters for all commands used to assemble the genome and construct the pangenome are available in figshare^43^.
